# Risk factors for bovine anthrax in Bangladesh, 2010–2014: a case-control study

**DOI:** 10.1017/S0950268820000576

**Published:** 2020-02-28

**Authors:** F. I. Rume, M. R. Karim, C. R. Ahsan, M. Yasmin, P. K. Biswas

**Affiliations:** 1 Department of Microbiology, Dhaka University, Dhaka, Bangladesh; 2 Department of Microbiology and Public Health, Patuakhali Science and Technology University, Barishal, Bangladesh; 3 Department of Medicine, Bangabandhu Sheikh Mujibur Rahman Agricultural University, Gazipur, Bangladesh; 4 Department of Microbiology and Veterinary Public Health, Chittagong (Currently, Chattogram) Veterinary and Animal Sciences University, Chattogram, Bangladesh

**Keywords:** Bangladesh, bovine anthrax, risk factors

## Abstract

A matched case-control study was conducted in Bangladesh by enrolling case smallholdings of cattle affected with anthrax in the period of October 2010 to December 2014. The cases were initially reported by mass media and/or in surveillance reports from authorities concerned in the country. In total, 43 case smallholdings were enrolled. For each case, a control was matched by similarity in herd-size and rearing of animals, selected from a distantly located (within 3–10 km) place but within the same sub-district of the case farm. Data collected by administering a prototype questionnaire were analysed by matched-pair analysis and multivariable conditional logistic regression. Out of the 43 smallholdings, 41 were located in three adjoining districts: Pabna, Sirajganj and Tangail, apparently forming a spatial cluster, could be termed ‘anthrax hot spot’ in Bangladesh. Sick animal on farm or a nearby farm slaughtered in the recent past (odds ratio (OR) 12.2, 95% confidence interval (CI) 1.6–93.4, *P* = 0.016)), history of heavy rains occurring in the last 2 weeks preceding an outbreak (OR 13.1, 95% CI 1.2–147.1, *P* = 0.037) and disposing of dead animal into nearby water body (OR 11.9, 95% CI 1.0–145.3, *P* = 0.052) were independent risk factors for anthrax in cattle in the country.

## Introduction

Anthrax is an acute infectious non-contagious zoonotic disease [1]. It is caused by a spore-forming bacterium, *Bacillus anthracis* which has a wide host range of animal species, including humans. Although anthrax is primarily considered a disease of herbivores, it is found in almost all warm-blooded animals with high mortality in several farms and wild animal species [2]. The clinical course of anthrax ranges from peracute to chronic [3]. Because of rapid course between clinical onset and death, vaccination against the disease is the first line of consideration for any control plan of anthrax in bovine animals. However, in developing countries such as Bangladesh, the vaccine supply is inadequate for the entire susceptible bovine population, and therefore, knowledge of risk factors are necessary for applying both vaccine and non-vaccine measures as cost-effective control strategies in resource-limited setting.

*B. anthracis* is usually transmitted to humans through contact with infected animals or contaminated animal products. The exposure pattern of the organism to humans results in three different forms of the disease: cutaneous (caused by penetration of the spores through micro-abrasions or cuts), gastrointestinal (caused by the consumption of infected meat) and inhalational anthrax (caused by inhalation of the spores). The cutaneous form predominates (constitutes >95% of all cases in an outbreak). In the absence of active surveillance for zoonotic diseases in Bangladesh, cases of animal anthrax are generally reported in mass media disclosing human cases attributed to butchering and slaughtering of clinically sick animals for meat. Such intermittent reports across the country are not expected to reveal the real burden of the disease in bovine animal population.

In Bangladesh there are eight administrative divisions and 64 districts. Unprecedented high numbers of human and animal anthrax cases were reported in the years 2009 and 2010 [4–6], recorded mostly from two districts, namely Pabna and Sirajganj in the division of Rajshahi. During the epidemic, a case-control study was conducted by enrolling 15 cases (occurred in July–September 2010) in that spatial zone [5]. Although assessing risks by including anthrax cases only from a narrower time and space might have had merits for a particular geographical area, the findings could not be generalised for the entire country. Besides, to the authors' knowledge, apart from that study, no risk factors for bovine anthrax have been reported locally from any other studies in the past. To fill the gap of knowledge with evidence-based research findings as part of designing non-vaccine preventive measures to control bovine anthrax, risk factors associated with the disease in the population covering wider time and space need to be identified. Because One Health approach is required to control anthrax more effectively, management of risk factors identified in cattle from broader time and space would eventually protect the animal and public health from the disease. Here, we describe the risk factors associated with anthrax affecting cattle in Bangladesh using a matched case-control study in a wider time space of about 4 years.

## Materials and methods

### Geography and cattle population statistics of Bangladesh

Bangladesh has the highest density of livestock (cattle, goats, sheep and buffaloes) in the world with an estimated 145 large ruminants per square kilometre of land compared with 90 in India and 20 in Brazil [7]. The total cattle and buffalo population of Bangladesh is about 26.22 million [8]; most of them are reared by villagers in smallholdings.

### Selection of case farm

We verified any reports/rumours on anthrax affecting humans and/or animals intermittently published from the Institute of Epidemiology, Disease Control and Research, the Department of Livestock Services, Bangladesh, National daily Newspapers and an electronic disease surveillance system for infectious diseases called ‘Programme for Monitoring Emerging Diseases-mail’ (ProMED-mail; http://www.promedmail.org/) in the period of October 2010 to December 2014, and physically visited the outbreak sites reported. Additionally, while visiting anthrax suspected smallholding in an area, any other smallholding(s) having a history of sudden death of ≥1 bovine animals in the recent past (30 days preceding of the date collection) with the onset of convulsions or falling down with or without previous reported fever were also visited and collected samples, such as remnants of organs and blood/carcass contaminated soils. Of the farms visited a case farm was enrolled in the study based on at least any one of the following three criteria:

1. At least one bovine animal from the farm/smallholding died with sudden onset of convulsions or falling down [9] and a large number of characteristic organisms (McFadyean reaction) were revealed in the blood films of the moribund or recently dead animals stained with 1% polychrome methylene blue [2]. Examination of blood films was performed at the nearby Field Disease Investigation Laboratory (FDIL) or at the Central Disease Investigation Laboratory (CDIL) in Dhaka (in Bangladesh one CDIL and eight FDILs provide livestock disease diagnostic services). Information confirming such examination was verified by official records kept at the FDIL or CDIL and interviewing the veterinarian involved with the case management.
2. A case farm was a farm with a history of slaughtering of a critically ill bovine animal for meat followed by typical anthrax lesions developed among those who were exposed to the suspected carcass, reported by mass media or the authority concerned in the country. Slaughter was performed by the Halal method [10]. Practices associated with slaughtering and butchering included skinning a carcass and processing and cooking meat.
3. Any remnant of organ and/or blood/carcass contaminated soil sample collected from a farm yard was tested positive for *B. anthracis* by the ground anthrax bacillus refined isolation (GABRI) method [11, 12]. And if the isolates resulted from initial screening by GABRI were found positive for the presence of *capC* (pXO2), *pagA* (pXO1) and *Dhp61* (BA_5345) genes by real-time polymerase chain reactions, as described previously [13, 14]. The GABRI method is more sensitive in revealing the presence of *B. anthracis* [11].

In total, 43 case farms were finally selected for this study.

### Selection of control farms

For each case farm, a control farm was selected within the same sub-district but located quite distantly (within 3–10 km) from the case farm representing the population at risk for anthrax. A control farm was matched with a case farm in terms of similarity in animal rearing and herd size, but a history of freedom from any febrile disease affecting its animals over the last 6 months from the date of the clinical onset of anthrax in the case farm, verified by interviewing the owner of the control farm. The freedom from of any febrile disease and distant location of a control farm, as mentioned, was to make sure that it was free from anthrax and potential exposure to any kind of materials coming from the case farm, respectively.

### Data collection

The data from each case and control farm were collected by a structured questionnaire designed to collect information on probable risk factors and the questionnaire was completed by the face-to-face interview with the farm owner. Population statistics of the case and control farms were noted, and global positioning system coordinates of the farms were recorded using personal navigators (eTrex Venture, Garmin, USA). A geographical information system programme (Arc GIS 10.2.2; Environmental System Research Institute, USA) was used to display the locations of the case farms enrolled in the study in a map of the country.

### Statistical analysis

The data were entered into a spreadsheet (Ms Excel 2000, Microsoft) (the spreadsheet is available on request). To estimate the strength and statistical significance of association between a risk factor and the disease, we applied the matched-pair (McNemar) test using GraphPad Software Quick Calcs (http:// www.graphpad.com/quickcalcs/McNemar1.cfm). An association was considered significant if a test had *P* ⩽ 0.05. The data from the spreadsheet were transferred to Stata 11 (StataSE) (Stata Corporation, USA) and in order to examine the independence of effects, multivariable conditional logistic regression was applied, using ‘clogit’ syntax. Any variables with *P* < 0.20 after the McNemar test were included in the multivariable conditional logistic regression analysis. The model of risk factors was constructed by backward selection applying the iterative maximum likelihood estimation procedure.

## Results

### Spatial distribution of case farms

**Fig. 1.**
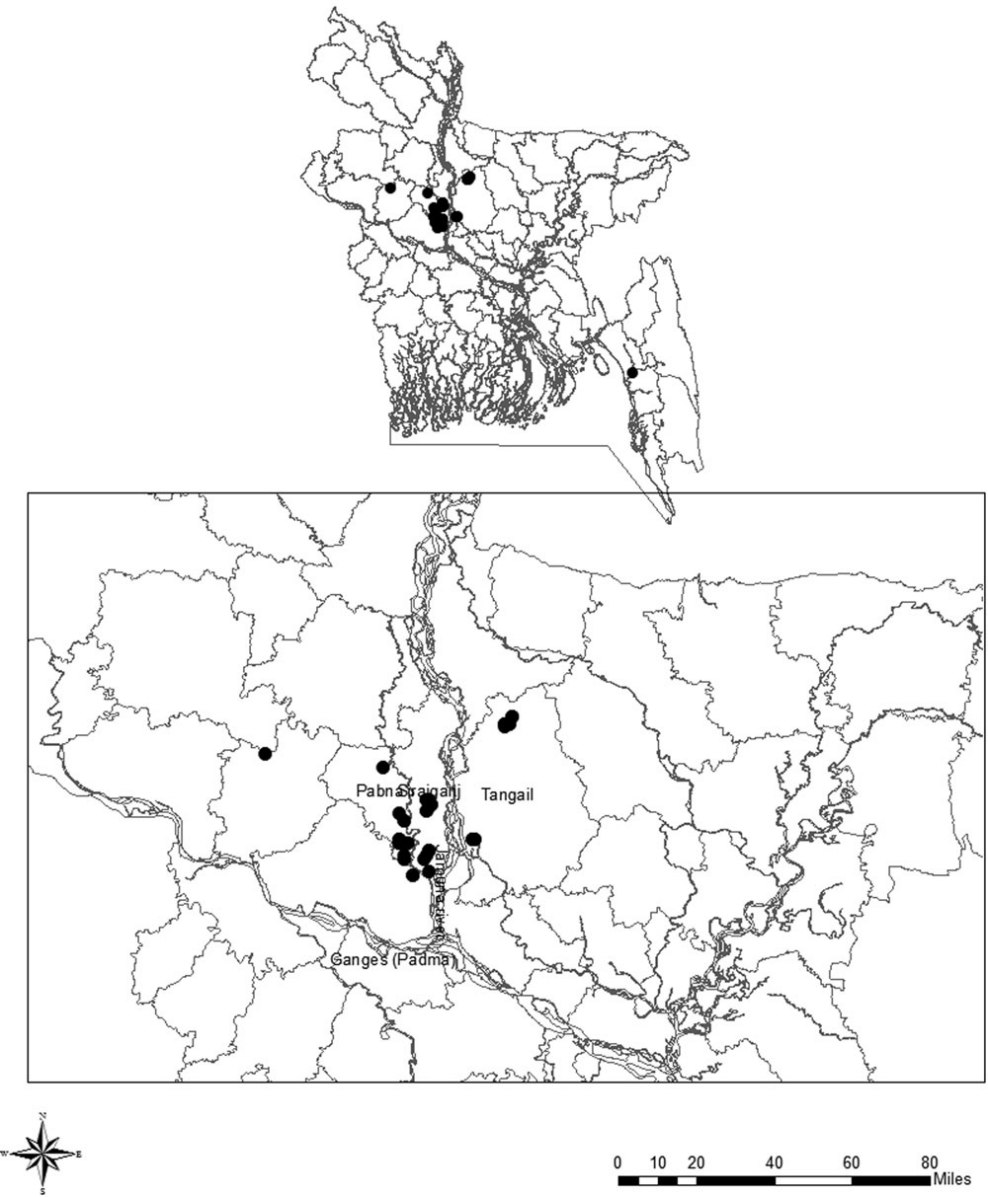
Geographical distributions of anthrax-affected cattle smallholdings observed in Bangladesh in October 2010–December 2015 that were enrolled in the study as the case farms; a closer view (at bottom) showing 41 of the 43 cases located in three adjoining districts: Pabna, Sirajganj and Tangail lying at the converging area of two rivers, Padma (Ganges) and Jamuna (western (main) branch of Brahmaputra).

The geographical locations of the case farms are shown in Figure 1 showing a map of Bangladesh. Except two, all (41/43) were clustered in three districts namely Pabna, Sirajganj and Tangail, situated in the adjoining areas where the two great trans-boundary rivers in South Asia Ganges (Padma in Bangladesh) and Brahmaputra (western branch called Jamuna in Bangladesh) have converged. The apparent clustering of the case farms however occupied seven out of the 30 sub-districts under the three districts mentioned. Due to very closer geographical positions, locations of some of the case farms appear to be overlapped, and thus are not well-distinctive in the figure. News on three outbreaks was not reported in any mass media or agency's reports. They were identified on the basis of the results of laboratory investigations done on the remnants of organ and/or soil samples collected from the farmyards where recent bovine animals had suddenly died and such events were reported to the first author from the local people while collecting information from the reported case farms in the affected villages.

### Population statistics of the case and control farms

Population statistics of the case and control farms are presented in Table 1. The median number of cattle observed on the day of survey in the case or in the control farms were four, ranging from 2 to 26 and 1 to 22 in the case and control farms, respectively.

**Table 1.** Population statistics of the case and control farms enrolled in the study for the assessment of risk factors associated with anthrax in cattle in Bangladesh, 2010–2014

Farm status	Animal No.				
	*N*	Max	Min	Mean	Median
Case	43	26	2	5.81	4
Control	43	22	1	5.35	4

### Risk factors

The variables assessed and the results of the matched-pair analysis are presented in supplementary file S1. Out of the variables assessed only three were significant at *P* < 0.05. Of them the factor, ‘Sick animal on farm or a nearby farm slaughtered in the recent past’ had the strongest point estimate of effect (odds ratio (OR) 14.0, 95% confidence interval (CI) 2.1–591.0; *P* = 0.002). According to the strength of point estimate, the other two risk factors were: ‘history of heavy raining occurred in the area in the last two weeks preceding the death of an animal in the farm’ (OR 11.0, 95% CI 1.6–473.5, *P* = 0.010) and ‘Dead animal disposed of into nearby water body’ (OR 8.0, 95% CI 1.1–354.0, *P* = 0.046).

After matched-pair analysis eight variables with *P* < 0.20 were considered for entering into the conditional logistic regression analysis and their effects in the initial model are displayed in Table 2. However, only three variables with *P* < 0.05 were retained in the final model as independent risk factors (Table 3). According to the probability of risk-association they were sick animal on farm or a nearby farm slaughtered in the recent past (OR 12.2, 95% CI 1.6–93.4, *P* = 0.016), history of heavy raining occurred in the last 2 weeks (13.1, 95% CI 1.2–147.1, *P* = 0.037) and disposing of dead animal into nearby water body (OR 11.9, 95% CI 1.0–145.3, *P* = 0.052).

**Table 2.** Multivariable analysis of risk factors associated with anthrax affecting cattle in smallholdings in Bangladesh (initial model)

Variable	OR	95% CI	*P* value
Flooded area	3.6	0.3–45.2	0.318
Half building/semi-paka housing	1.5	0.2–16.0	0.712
Free grazing	1.5	0.2–13.7	0.730
Feeding animals with water hyacinth	10.3	0.3–371.4	0.202
Sick animal on farm or a nearby farm slaughtered in the recent past	15.6	1.5–159.7	0.021
Disposing of dead animal into nearby water body	33.0	0.9–1149.7	0.053
History of heavy rains occurring in the last 2 weeks preceding an outbreak	23.1	0.9–600.5	0.059
Anthrax vaccination during disease outbreak	4.3	0.4–44.0	0.224

OR, odds ratio; CI, confidence interval.

Logistic regression; initial model with eight variables entered; *χ*^2^(8) for likelihood ratio test 35.21; *P* < 0.001; No. of observation = 86.

**Table 3.** Multivariable analysis of risk factors associated with anthrax affecting cattle in smallholdings in Bangladesh (final model)

Variable	OR	95% CI	*P* value
Sick animal on farm or a nearby farm slaughtered in the recent past	12.2	1.6–93.4	0.016
Disposing of dead animal into nearby water body	11.9	1.0–145.3	0.052
History of heavy rains occurring in the last 2 weeks preceding an outbreak	13.1	1.2–147.1	0.037

OR, odds ratio; CI, confidence interval.

Logistic regression; *χ*^2^(3) for likelihood ratio test 27.44; *P* < 0.001; No. of observation = 86.

## Discussion

An unprecedented epidemic trend of anthrax outbreaks affecting both bovine animals and humans were recorded in the districts of Pabna and Sirajganj in monsoon months of 2 consecutive years, 2009 and 2010 [4–6]. Any such temporal peak of outbreaks was not reported in the period of the present study, October 2010 to December 2014 in the country. Following the mass media and/or available surveillance reports, we were able to identify only 40 outbreaks of anthrax across the country, and the other three were identified by testing remnants of organ and/or soil samples collected from adjacent farms which reportedly had a history of sudden mortality. Such a low number of outbreaks over a long period of observation indicate that anthrax outbreaks in animals in Bangladesh are predominantly reported when humans are also affected as a result of slaughtering and butchering of clinically diseased animals for meat. This practice is against universally accepted public health recommendations but farmer preferences are inclined to minimise financial losses of a valuable animal by selling its meat at a cheaper price. In African countries socio-cultural practices at the community level such as slaughtering of sick animals or salvage butchering of dead animals, and eating or handling the meat from these infected animals, contribute to the recurrence of human anthrax cases [15–17]. Culturally and in religious viewpoint, meat from dead animals is not eaten in Bangladesh. However, clustering of 41 out of the 43 outbreaks in only three adjacent districts Pabna, Sirajganj and Tangail lying at the merging site of the rivers Padma and Jamuna suggests that the area may remain as an ‘anthrax hotspot’ in Bangladesh, as demonstrated in the years 2009 and 2010 [4–6].

Intermittent mass media reports, as rumours, on anthrax affecting bovine animals in Bangladesh do not disclose its true prevalence, which might be much higher because a retrospective study conducted on veterinary field data submitted from the entire country during the period 1 January 2010 to 31 December 2012 revealed 5937 animal cases of anthrax [18]. The shortcoming of these data used in the article referred was that the occurrences of the cases were not confirmed by laboratory testing. Thus, cases of bovine anthrax occurred yearly meeting any of the three criteria set for this study were very limited.

During the peak of anthrax outbreaks in July–September 2010 a case-control study was conducted previously by enrolling only 15 cases [5] and the study identified ‘Feeding animals with water hyacinth (*Eichhornia crassipes)*’ an independent risk factor. In the present study, on the results of univariable analysis (Supplementary Table S1) its association with anthrax was weakly significant (*P* = 0.131), qualifying the variable to be entered into the multivariable analysis. Probably, due to the small sample size, it was not found an independent risk factor in the end. However, the present study identified three new risk factors.

Being fearful of economic loss, farmers sometimes slaughter their severely clinically affected bovine animals for selling of meat unknowing the danger of transmission of *B. anthracis* to humans. Such slaughtering places, which are usually the farmyards, are not properly decontaminated, but rinsed with water to remove the blood and slaughter-associated wastes, and butchering wastes are often disposed of into nearby ditches, water bodies or on open fields, contaminating the environment [19]. *B. anthracis* can produce spores in the said contaminated places and live in this dormant stage for years together. While feeding on the recently contaminated patches of grass/vegetation on such farm yards other animals of the same farm or nearby farms can be infected, could be the probable reason of finding ‘Sick animal on farm or a nearby farm slaughtered in the recent past’ an independent risk factor. In a previous study, we identified the same sub-genotype (lineage A major subgroup A. Br 001/002 GT2/Ban subGT5) of the organism from some remnants of bone and the soil samples from the same farmyard of a case farm [12]. In North Dakota, USA, death of an animal on a neighbouring premise was found to be a significant predictor of anthrax occurrence on a premise [20].

It should be again noted that a control farm was matched with its case farm from a distantly located area of the case farm but within the same sub-district. This was for ensuring the freedom of the control farm from being exposed to any kind of environmental materials contaminated with emanations of the case farm. In this study, heavy rain was described as intense rainfall in a short period of time. Most cases enrolled in the study occurred in the rainy season (July–October). Heavy rainfall variability in short time periods within an area of a sub-district is although not a frequently observed phenomenon, but can be seen in monsoon season in Bangladesh. The results of this study showed that this variation occurred in about 25% of the cases, indicating it an independent risk factor for bovine anthrax in Bangladesh. Heavy rainfall washes off the topsoil exposing the anthrax bacilli spores underneath that could be accumulated on certain spots on the ground through water runoffs. Cattle could be infected by feeding soil-adhered new fresh shoots of grass grown on such places and their roots containing an infective dose of anthrax bacilli spores [21].

Locations of farms in a river basin and flooding were suspected of having contributory roles in anthrax outbreaks in North Dakota, USA and in Sweden [20, 22]. In a previous study in Bangladesh, it was found that farmers disposed of dead animals into nearby water [9]. Dumping carcasses in the water may lead to the contamination of the water. Offering Muddy water collected from such water bodies could carry anthrax bacilli spores. Spores of *B. anthracis* have a high buoyant density, which provides an opportunity for them to adhere to vegetation as the vegetation resurfaces during evaporation [21]. Floating and decomposed carcass could also contaminate the surrounding vegetation which could also contribute to infection, if animals fed on such vegetation. Disposal of a dead animal into nearby water body in the area of a case farm could indicate that such a carcass floating on nearby water would be a predictor for seeing the emergence of new anthrax case in the area.

Verification records on anthrax vaccinations in the case and control farms were absent, although during the interviews owners of 11% (5/43) and 14% (6/43) case and control farms, respectively, responded that their animals had been vaccinated against the disease. However, they could not recall the exact dates of the vaccinations. The Sterne strain of *B. anthracis* is used for immunisation and should be administered to livestock in a dose containing up to 10 million viable spores [23]. Animals vaccinated >6 months ago might not retain sufficient immunity because Sterne strain vaccine induces immunity that typically lasts for just under 1 year [24]. A 6-month interval between two doses of vaccine against anthrax might be ideal for the anthrax-prone area to protect the susceptible animals from the disease [24].

One of the limitations of this study was the smaller sample size of the cases, although we included all the bovine farms that met any of the three selection criteria in a study period of 4 years, October 2010 to December 2014 in the country. This smaller sample size was also the reason for reporting very wide confidence intervals from point estimation of a risk factor assessed. However, on the background of an extremely low level of validly reported and verified cases and the importance of information on risk factors for anthrax to recommend non-vaccine driven intervention along with vaccination, the logical grounds were considered to justify carrying out the study.

Insufficient vaccine production (3.8 million vaccine doses against approximately 48.7 million ruminant population) [25] might be the reason for weak vaccine coverage against anthrax even in the anthrax hotspot in Bangladesh. The mitigations of the risk factors identified would be helpful in the country to enhance non-vaccine control measures. Awareness building among the farmers by the field veterinarians, their supporting staff and the non-governmental workers on the deadly threats being posed to their own lives and livestock from slaughtering of clinically sick animals is important to stop this unsafe practice. After a heavy rainfall, animals should not be allowed to feed on freshly shoots of grass, particularly grown on farmyard and grazing fields where animals were recently slaughtered or dead animals were disposed of. Any dead animal must not be disposed of into open water or on the field, but buried on a dry land sufficiently away from any water body following deep burial method under veterinary supervision. In conclusion, practice of the non-vaccination measures aiming at mitigating the three risk factors identified along with the routine vaccination would be more effective in controlling bovine anthrax in Bangladesh.

## Supplementary material

For supplementary material accompanying this paper visit https://doi.org/10.1017/S0950268820000576. click here to view supplementary material
