# Supplementary material for: Risk factors for bovine anthrax in Bangladesh, 2010–2014: a case-control study
Source: Epidemiol Infect. 2020 Feb 28;148:e67. doi: 10.1017/S0950268820000576 (PMC7118724; doi:10.1017/S0950268820000576)
Supplement: Supplementary file 1 [file S0950268820000576sup001.docx]

**Supplementary Table S1. Matched-pair analysis of risk factors for anthrax in cattle in Bangladesh (N=43 pairs: 43 case farms, 43 control farms, 2010 - 2014)**

| Risk factor | | N(+/-) | N(-/+) | OR (95% CI)* | P value |
| --- | --- | --- | --- | --- | --- |
| Farmer’s education | Primary | 6 | 9 | 0.7 ( 0.2- 2.1 ) | 0.606 |
|  | Secondary | 10 | 7 | 1.4 (0.5-4.4) | 0.628 |
|  | Higher Secondary | 0 | 1 | - | 1.000 |
|  | Above | 1 | 0 | - | 1.000 |
| Farmer’s occupation | Agriculture | 7 | 8 | 0.9 (0.3-2.8) | 1.000 |
|  | Animal Husbandry | 0 | 2 | - | 0.480 |
|  | Service | 3 | 1 | 3.0 (0.2-157.5) | 0.617 |
|  | Business | 6 | 5 | 1.2 (0.3-5.0) | 1.000 |
| Spatial | Plain land | 1 | 4 | 0.3 (0-2.5) | 0.371 |
|  | Coastal area | 0 | 0 | - | - |
|  | Marshy land | 0 | 0 | - | - |
|  | Flooded area | 10 | 4 | 2.5 (0.7-11.0) | 0.181 |
|  | Drought area | 0 | 0 | - | - |
|  | Hilly area | 0 | 0 | - | - |
| Location of the farm | Near by a road | 1 | 0 | 0 | 1.000 |
|  | Near by a river | 2 | 3 | 0.7 (0.1-5.8) | 1.000 |
| Housing system | Old pattern/ kacha | 6 | 4 | 1.5 (0.4-7.2) | 0.752 |
|  | Half building /semi paka | 2 | 8 | 0.3 (0-1.3) | 0.114 |
|  | Building/paka | 0 | 1 | 0 | 1.000 |
| Floor type | Animals kept tethered on wet muddy area | 6 | 5 | 1.2 (0.3-5.0) | 1.000 |
|  | Animal kept tethered on dry place | 5 | 6 | 0.8 (0.2-3.3) | 1.000 |
| Feeding system | Mostly stall feeding | 3 | 2 | 1.5 (0.2-18.0) | 1.000 |
|  | Some grazing opportunity (only at homestead) | 2 | 2 | 1.0 (0.1-13.8) | 0.617 |
|  | Free grazing | 9 | 3 | 3.0 (0.8-17.2) | 0.149 |
|  | Feeding animals with uprooted and unwashed grass | 9 | 9 | 1.0 (0.4-2.8) | 0.814 |
|  | Grazing field recently flooded | 2 | 1 | 2.0 (0.1-118.0) | 1.000 |
|  | Dry grazing field | 0 | 0 | - | - |
|  | Feeding animals with water hyacinth | 6 | 1 | 6.0 (0.67-276.0) | 0.131 |
|  | Feeding animals with feed added with bonemeal | 0 | 0 | - | - |
|  | Grass collected from places where animal carcasses are disposed of in dry season | 0 | 0 | - | - |
| Source of drinking water | Canal/pond | 1 | 1 | 1.0 (0-78.5) | 0.480 |
|  | Clean/ muddy | 1 | 0 | 0 | 1.000 |
|  | River | 0 | 0 | - | - |
|  | Clean/ muddy | 0 | 0 | - | - |
|  | Tube- well | 1 | 1 | 1.0 (0-78.5) | 0.480 |
|  | Supply water | 0 | 1 | 0 | 1.000 |
| Sick animal on farm or a nearby farm slaughtered in the recent past | - | 14 | 1 | 14.0 (2.1 -591.0) | 0.002 |
| Types of disposal of dead animal in the area | Buried | 24 | 0 | 0 | 0 |
|  | Burning | 0 | 0 | 0 | 0 |
|  | Into nearby water body | 8 | 1 | 8.0 (1.1 – 354.0) | 0.046 |
| Proximity of burial place | Near to household | 4 | 0 | 0 | - |
|  | Far from household | 26 | 0 | 0 | 0 |
| History of heavy raining occurred in the last 2 weeks preceding the death of an animal in the farm | - | 11 | 1 | 11.0 (1.6 – 473.5) | 0.010 |
| History of recently increased blood sucking insects population (HBP) | - | 1 | 4 | 0.3 (0-2.5) | 0.371 |
| Vaccination time | Before outbreak | 5 | 6 | 0.8 (0.2-3.3) | 1.000 |
|  | During outbreak | 10 | 4 | 2.5 (0.7-10.9) | 0.181 |

OR, Odds ratio; CI, confidence interval; N (+/-), number of exposed case and unexposed control pairs; N (-/+), number of unexposed case and exposed control pairs.

* Matched-pair analysis using McNemar’s test.
